# A Retina Inspired Model for Enhancing Visibility of Hazy Images

**DOI:** 10.3389/fncom.2015.00151

**Published:** 2015-12-22

**Authors:** Xian-Shi Zhang, Shao-Bing Gao, Chao-Yi Li, Yong-Jie Li

**Affiliations:** ^1^Key Laboratory for Neuroinformation of Ministry of Education, School of Life Science and Technology, University of Electronic Science and Technology of ChinaChengdu, China; ^2^Center for Life Sciences, Shanghai Institutes for Biological Sciences, Chinese Academy of SciencesShanghai, China

**Keywords:** haze removal, retina inspired model, retinal ganglion cell, non-classical receptive field, disinhibitory effect

## Abstract

The mammalian retina seems far smarter than scientists have believed so far. Inspired by the visual processing mechanisms in the retina, from the layer of photoreceptors to the layer of retinal ganglion cells (RGCs), we propose a computational model for haze removal from a single input image, which is an important issue in the field of image enhancement. In particular, the bipolar cells serve to roughly remove the low-frequency of haze, and the amacrine cells modulate the output of cone bipolar cells to compensate the loss of details by increasing the image contrast. Then the RGCs with disinhibitory receptive field surround refine the local haze removal as well as the image detail enhancement. Results on a variety of real-world and synthetic hazy images show that the proposed model yields results comparative to or even better than the state-of-the-art methods, having the advantage of simultaneous dehazing and enhancing of single hazy image with simple and straightforward implementation.

## Introduction

The necessary processing of visual information already begins in the eye, the very first stage of the visual system. Increasing evidence suggests that the mammalian retina seems far smarter than scientists have believed so far (Gollisch and Meister, 2010; Lee et al., 2010; Masland, 2012). The retina is a neural circuit composed of at least 50 clearly distinct cell types (Joselevitch, 2008). These cells form various retinal subsystems that serve a diverse set of specific tasks, e.g., light adaptation and image sharpening. Together, these retinal neurons and their coding strategies enable the visual system to perform well by adapting to the complicated environments, e.g., with changing air conditions.

**Figure 1:**
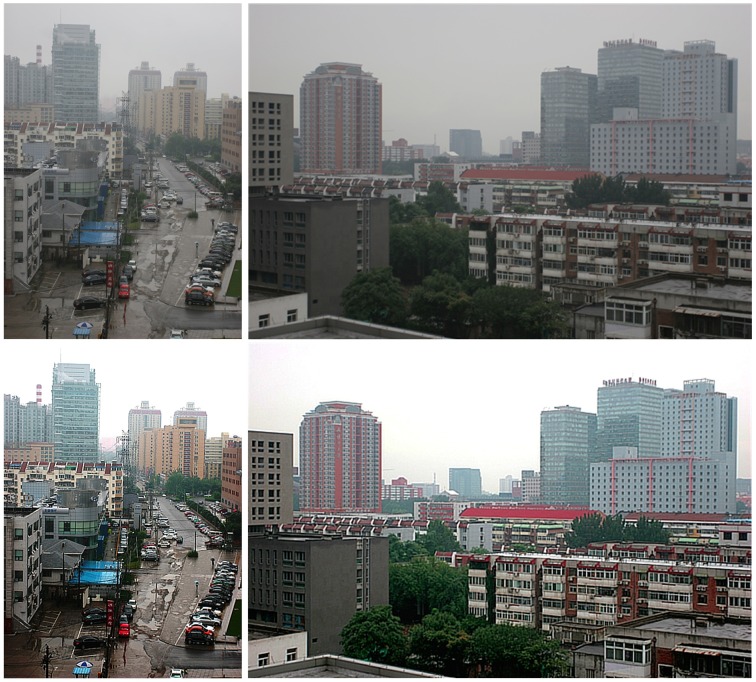
**Hazy images (top) and the images after haze removal by the proposed method (bottom)**.

From the point of view of engineering, many computer vision applications expect clear input images with high-contrast details. However, this situation is not always true in practical scenarios. Due to the presence of aerosols such as dust, mist and water droplets in the atmosphere, the reflected light from the object surface has already been scattered before it reaches the camera. As shown in Figure 1, this light-scattering phenomenon consequently results in the contrast reduction and color fading, which eventually cause the definition decrease in the captured images. This image degradation annoys not only computer vision applications but also consumers and commercial photographs, and therefore, image dehazing and visibility enhancing have become more and more important in this digital age.

Haze removal is a challenging task because the haze condition is always unknown. Most existing methods use additional information to solve this ill-posed problem. For example, Schaul et al. combine the hazy image with its near-infrared version (Schaul et al., 2009). Depth-based methods assume that the depth information of the image is available or is accessible by the three-dimensional (3D) geometrical model of the scene and then use it to enhance the image (Tan and Oakley, 2000; Narasimhan and Nayar, 2003b; Hautière et al., 2007; Kopf et al., 2008). Although these methods can enhance the visibility of hazy images, they cannot be applied when such additional information is unavailable. There are also some methods that use two or more images of a same scene to estimate the haze (Nayar and Narasimhan, 1999; Narasimhan and Nayar, 2000, 2003a; Schechner et al., 2001; Shwartz et al., 2006; Namer et al., 2009; Treibitz and Schechner, 2009). In general, these methods can remove haze well but require multiple images. For a single image, haze removal methods based on some priors have been developed. For example, Tan (2008) assumes that a haze-free image should have higher contrast than the input hazy image, based on which the haze is removed by maximizing the local contrast of the input hazy image. Tarel et al. develop a real-time haze removal method (Tarel and Hautiere, 2009), also based on the information of local contrast, by assuming that the atmospheric veil is smooth at most of the time and taking the percentage between the local standard deviation and the local mean of the whiteness as the air light. Fattal (2008) assumes that the transmission and the surface shading in a single input image are locally uncorrelated, and then eliminates the scattered light by estimating the optical transmission in hazy scenes. Kratz and Nishino (2009) try to estimate both the scene albedo and depth, which contain valuable structural information of haze. Ancuti et al. (2011) assume that the distance between the observer and the scene objects is highly correlated with the contrast degradation and color fading, and then detect haze quickly by a comparison of the hue values in the input image and its “semi-inversed” version. Dark channel prior based methods (He et al., 2009, 2011) assume that the pixels in the dark channel of haze-free images are close to zero, while in hazy images these pixels obtain higher intensity from the airlight, and therefore utilizing these pixels can accurately estimate the haze transmission. Many recent progresses improve the dark channel based methods by replacing matting with other filters (Yu et al., 2010; Gibson et al., 2012) or adding new constraints (Tarel et al., 2012; Caraffa and Tarel, 2013; Meng et al., 2013). More recently, Tang et al. (2014) propose a learning framework to combine different haze-relevant features and provide flexibility for different specific situations.

Although there are remarkable progresses in single image haze removal, the problem is that most of these methods require additional information or prior assumptions. However, in different real-world images, the information may miss, or the assumptions may fail, and then the methods based on them would perform worse than expected. For example, when the scene objects are similar to the atmospheric light, the dark channel of the scene radiance has bright values near such objects, which means that the dark channel prior is invalid, and as a result the haze layer will be overestimated (He et al., 2011).

Along another line, attempts that follow the information processing mechanisms of the human visual system (HVS) seems to be a promising route to address this problem, inspired by the amazing ability of HVS to achieve stable perception under varying natural light environments (Foster, 2011). Models inspired by HVS have succeeded in many fields, such as face recognition (Vu and Caplier, 2009), boundary detection (Yang et al., 2013, 2015), key point descriptor (Alahi et al., 2012), color constancy (Spitzer and Semo, 2002; Spitzer and Barkan, 2005; Gao et al., 2013, 2015), multi-resolution image-fusion (Ghassemian, 2001) and image enhancement (Land and McCann, 1971; Land, 1986; Jobson et al., 1997; Rahman et al., 2004), with robust performance under varying conditions. Representatively, the models based on the famous Retinex theory, which approximates the spectral properties of object surfaces by the ratio of the reflected light in this area to others (Land and McCann, 1971; Land, 1986; Jobson et al., 1997; Rahman et al., 2004), can enhance hazy images with (Xie et al., 2010; Nair et al., 2014) or without priors (Woodell et al., 2005; Rajput and Rahman, 2008; Zhou and Zhou, 2013). Taken inspiration mainly from the color perception behavior of human in psychophysical experiments, Retinex theory has not yet clarified whether the formation of lightness images and their comparison occur in the retina, the cortex, or the both (Foster, 2011).

Different from the Retinex based models, our proposed model enhances hazy images by simulating the underlying mechanisms at the specific level of retina. In particular, the proposed model includes the processing inspired by the physiological findings that the receptive field (RF) surround of retinal ganglion cell (RGC) consists of many inhibitory subunits (or subfields), and the inhibitory interactions among them lead to a disinhibitory effect, which means an adaptive reduction of the surround inhibition to the RF center (Li and He, 1987; Li et al., 1991, 1992; Li and Li, 1994; Qiu and Li, 1995; Li, 1996). In addition, the information processing along the ON and OFF pathways is combined. This proposed model directly enhances the input hazy image, without requiring to first estimate a transmission map as did in most dehazing models. The novelty of the proposed model lies in its ability of simultaneous haze removing and detail enhancing of single image due to the specific mechanisms of different retinal sub-layers. The contribution of this work is not only an efficient way to enhance hazy images for computer vision applications, but also a computational description about the possible retinal mechanisms of image enhancement in biological vision.

## Model

### General description

**Figure 2:**
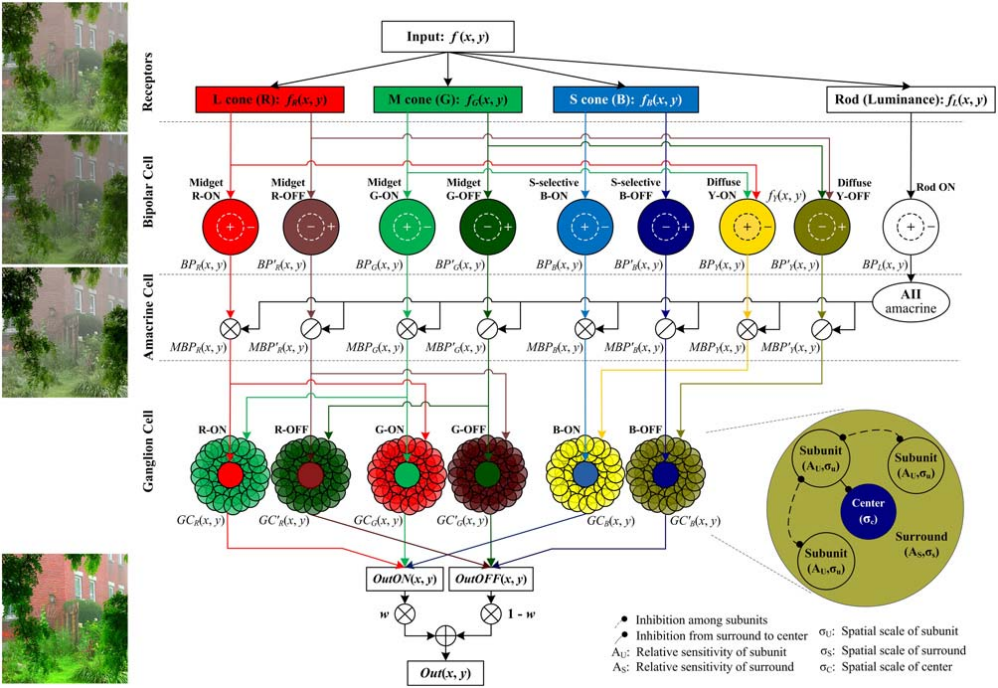
**The structure of the proposed retina-based model**. The R, G, B, and brightness components of the input color image are respectively sent into the corresponding photoreceptors. Then the outputs of cone photoreceptors are transmitted via cone bipolar cells, the RF of which is a difference of Gaussian function, to the RGCs, the RF of which consists of a small excitatory center and a relatively large inhibitory surround (also named the non-classical receptive field, nCRF). The surround is composed of many inhibitory subunits, which first inhibit each other, and then inhibit the center. The outputs of rod bipolar cells modulate the outputs of cone bipolars. From top to bottom, the image patches listed on left side are respectively the input image, the output of the bipolar cells, the bipolar output modulated by AII amacrine cells, and the final output the ganglion cells. Note that in primates, the densities of M and L cones are typically higher than S cone density by 20-fold (Euler et al., 2014), so the signals sent into the cone type-non-selective diffuse bipolar cells are mainly R and G components, and here we omit the B component sent into the diffuse bipolars for clarity and simplicity. *w* is a weight controlling the relative contributions of the ON and OFF pathways, and we set *w* = *f*_*L*_(*x, y*) in this work (see details in the text).

The proposed method follows the color processing mechanisms in the retina (Figure 2). The single hazy image is the input and the enhanced image is the output.

Retinal information processing begins with the sampling of rod and cone photoreceptors. The red (R), green (G), and blue (B) components of the input color image are responded respectively by long-, medium-, and short-wavelength cone photoreceptors (i.e., L, M, and S cones) of retina, while the brightness of the dim regions in the input color image is sensitively responded by rod photoreceptors (Masland, 2012). The photoreceptor activities are adjusted and transmitted to the RGCs via several retinal sub-layers like horizontal cells, bipolar cells and amacrine cells. In particular, the cone signals are then transmitted by ON- and OFF-type cone bipolar cells, whereas the rod signals are transmitted only by the ON-type rod bipolars, supported by the current biological evidence that unlike the cones, the rod system in the cat, monkey, and human has only ON-type rod bipolars (Schiller, 2010). In the ganglion-cell layer, the output layer of the retina, color signals are processed by the RGCs with single-opponent receptive field (RF), which receives opponent stimuli in its excitatory center and inhibitory surround from two (or more) different cones (Conway et al., 2010). The RF surround of a RGC is physiologically supposed to comprise many inhibitory subunits, each of which is first inhibited by its neighboring subunits (i.e., disinhibit), and then inhibits the neuronal response elicited by the RF center.

### Visual processing in bipolar cells

After the light absorption by photoreceptors, bipolar cells transmit the neuronal activities to RGCs. Continuous glutamate release from photoreceptors is suppressed by the light. Thus, bipolar cells that express sign-conserving ionotropic glutamate receptors are depolarized in the dark while bipolar cells that express sign-inverting metabotropic glutamate receptor are depolarized in the light. Differed in terms of response polarity, there are two major classes of bipolar cells: ON bipolar cells, which response to light on-set, and OFF bipolar cells, which response to light off-set (Schiller, 2010). Beside the RF center, an inhibitory surround of bipolar cells has been identified more than 40 years (Werblin and Dowling, 1969b). The RF of most bipolar cells consists of two regions: a smaller excitatory center and a lager inhibitory annular surround (Werblin and Dowling, 1969a; Kaneko and Tachibana, 1983), which is commonly described by the “difference of Gaussian” (DOG) model (Rodieck and Stone, 1965; Enroth-Cugell and Robson, 1966). The neurons with such RF type can transmit high-acuity and chromatic opponent signals. Although the cellular mechanisms and neural circuitry are not totally clear, many evidences support that horizontal cells mainly contribute to this surround antagonism (Thoreson and Mangel, 2012).

Based on the dendritic morphology, primate cone bipolar cell types have been divided into three groups: *diffuse bipolar cells, S-cone-selective bipolar cells*, and *midget bipolar cells*. Diffuse bipolar cells non-selectively contact between four and fifteen neighboring cones of various types. Because in primates, the combine density of M- and L-cones is typically higher than S-cone density by 20-fold, the signals of diffuse bipolar cells tend to be chromatically biased toward yellow (Euler et al., 2014). For better understanding, we also call these diffuse bipolar cells the *yellow-sensitive bipolar cells* in this paper. Combined with S-cone-selective bipolar cells, diffuse bipolar cells contribute to blue-yellow color vision. Near the fovea, a midget bipolar cell receives direct input from just one cone and in turn transfers the signal to one midget ganglion cell. The midget bipolar cells carry chromatic signals and are considered to be the basis for red-green color vision (Euler et al., 2014). The majority of cones in central retina connect with at least one ON bipolar cell and one OFF bipolar cell, which in turn connect respectively with ON and OFF retinal ganglion cells (Schiller, 2010).

There are two pathways in which cone bipolar cells carry chromatic information to RGCs. First, the cone type unselective pathway, in which a midget bipolar cell randomly contacts a single cone and feed into a single midget RGC, carries red-green information. Second, blue-yellow information is carried by the cone type selective pathway. In this way, the fact that blue-ON-yellow-OFF RGCs differentially pool signals from S-cone-selective ON and diffuse OFF bipolar cells is well-known, but where blue-OFF-yellow-ON RGCs inherit the blue-OFF signal from is still controversial. Some former researches report that the blue-OFF signal is from S-cone-selective ON bipolar cells via a sign-inverting small field amacrine cell (Chen and Li, 2012), while recent anatomical and physiological evidences support that it is from S-cone-selective OFF bipolar cells directly (Mills et al., 2014).

Given an input image *f*_*c*_(*x, y*), *c* ∈ {*R, G, B*} normalized within [0, 1] by dividing each channel with the maximum intensity across three channels, the three components are respectively sampled by three cone types of cones, and the luminance component *f*_*L*_(*x, y*) described by Equation (1) is sampled by the rods

(1) $$f_{L}(x,y)=(f_{R}(x,y)+f_{G}(x,y)+f_{B}(x,y))/3.$$

Then, the signals sampled by the photoreceptors are further sent into the bipolar cells. For clarity, we use *f*_*Y*_(*x, y*) to represent the combined signals of R, G, and B sent into the diffuse yellow (Y)-sensitive bipolar cells. By ignoring the quite less input from B channel based on the physiological evidence (Euler et al., 2014), *f*_*Y*_(*x, y*) can be simply computed as

(2) $$f_{Y}(x,y)=(f_{R}(x,y)+f_{G}(x,y))/2.$$

As indicated in Figure 2, the color components sampled by cone photoreceptors are further processed by the R, G, B, and Y-sensitive bipolar cells of both ON and OFF types, and in contrast, the luminance component sampled by rods is processed by the rod bipolar cells of only ON type (Schiller, 2010). In a general form, we denote the outputs of ON bipolar cells as *BP*_*c*_(*x, y*), *c* ∈ {*R, G, B, Y, L*} and the outputs of OFF bipolar cells as $BP_{c}^{\prime }\left(x,y\right),\;c\in \left\{R,G,B,Y\right\}$, which are computed as

(3) $$BP_{c}(x,y)=f_{c}(x,y)⊗\left(g(x,y;\sigma_{cen})-k\;\cdot \;g(x,y;\sigma_{sur})\right)$$

(4) $$BP_{c}^{\prime }(x,y)=f_{c}^{\prime }(x,y)⊗\left(g(x,y;\sigma_{cen})-k\;\cdot \;g(x,y;\sigma_{sur})\right)$$

where ⊗ is a convolution operator. $f_{c}^{\prime }(x,y)=1-f_{c}(x,y)$ is the input sent into the OFF bipolar cells. *k* represents the sensitivity of the inhibitory annular surround, σ_*cen*_ and σ_*sur*_ are respectively the standard deviations of Gaussian shaped RF center and its surround, which are experimentally set to be 0.5 and 1.0, respectively in this work. *g*(*x, y*; σ) is a two-dimensional (2D) Gaussian function written as

(5) $$g(x,y;\sigma )=\frac{1}{2\pi \sigma^{2}}\exp\left(-(x^{2}+y^{2})/(2\sigma^{2})\right).$$

The ON-type rod bipolar cells send their outputs *BP*_*L*_ (*x, y*) to the specific AII type of amacrine cells (Schiller, 2010). The AII amacrine cells modulate the activities of ON cone bipolar cells via sign-conserving gap junctions and OFF cone bipolar cells via inhibitory synapses (Lee et al., 2010). By this way, rod bipolar cells finally excite the ON RGCs and inhibit the OFF RGCs indirectly. Based on this biological fact, the modulated responses of ON and OFF bipolar cells, *MBP*_*c*_(*x, y*) and $MBP_{c}^{\prime }\left(x,y\right)$, are respectively given by

(6) $$MBP_{c}(x,y)=BP_{c}(x,y)\;\cdot \;\left(\varepsilon +BP_{L}(x,y)\right)$$

(7) $$MBP_{c}^{\prime }(x,y)=BP_{c}^{\prime }(x,y)/\left(\varepsilon +BP_{L}(x,y)\right)$$

where ε is a constant, which is set to be 0.5 to avoid being divided by zero and also to keep the multiplier and divisor to be around 1.0 for easy control in the later use. Note that *BP*_*c*_(*x, y*) and $BP_{c}^{\prime }\left(x,y\right)$ have been rectified by setting the negative values to zero and then normalized respectively to [0, 1] by dividing each channel with the maximum intensity across three channels.

It is clear that from Equations (6) and (7), with the modulation of AII amacrine cells that respond to the luminance component of scene (i.e., *BP*_*L*_(*x, y*)), the outputs of most cone ON bipolar cells (i.e., *BP*_*c*_(*x, y*), *c* ∈ {*R, G, B, Y*}) will be amplified in the brighter regions eliciting higher *BP*_*L*_ (*x, y*), and in contrast, the outputs of most cone OFF bipolar cells (i.e., $BP_{c}^{\prime }\left(x,y\right),\;c\in \left\{R,G,B,Y\right\}$) will be enhanced in the darker regions eliciting lower *BP*_*L*_(*x, y*). This will be further demonstrated in the section of *Experiments*.

### Disinhibition in RGCs

Retinal ganglion cells (RGCs) receive multiple cone signals transmitted via bipolar cells (and other cells) and compare them with the color-opponent mechanism. Like bipolar cells, the RF of most RGCs also consists of a smaller excitatory center and a larger inhibitory annular surround, and chromatically single-opponent RGCs receive inputs of different cone types within these two different RF regions (Conway et al., 2010). Although the RF center of a RGC, whose diameter is larger than a single cone because of physiological optics, a point spread function of the eye exceeds the size of a single cone in the fovea, is created by direct inputs from a single bipolar cell which contacts an individual cone, the synaptic pathways that create the opponent RF surround remain controversial (Lee et al., 2010). There are two main hypothesizes, i.e., unselective-connection and selective-connection. The unselective-connection hypothesis predicts that mixed cone inputs from non-selective horizontal cells to the surround lead to the opponency. In this hypothesis, color opponency is a product of interaction created by the horizontal cells and arises by subtracting individual cone forming center with all cones feeding in surround. This hypothesis is supported by some recently researches in the peripheral retina (Field et al., 2010; Crook et al., 2011). However, there are more direct physiological evidences supporting the hypothesis that the opponent surround is the result of cone specific or partially selective connection with bipolar cells (Martin et al., 2001; Reid and Shapley, 2002; Buzás et al., 2006; Sun et al., 2006; Lee et al., 2012). In this work we consider three types of single-opponent ON RGCs with selective connections: L/M, M/L, S/(L+M), which means that the firing rate of a RGC increases with the activation of one cone type (e.g., L or R) and decreases with the activation of a different cone type (e.g., M or G). In the following, we will use R, G, and B for short to denote respectively the above three single-opponent channels. In addition, physiological experiments have observed a secondary gentle rise in the neuronal response of some RGCs when the stimulus was further extended far beyond the RF center, which indicates a disinhibitory effect contributed by the extensive surround (Li and He, 1987; Li et al., 1991, 1992; Li and Li, 1994). The RF surround with disinhibitory effect (also called the non-classical receptive field, nCRF) was presumed to comprise many inhibitory subunits, which first inhibit each other, and then inhibit the RF center (Qiu and Li, 1995; Li, 1996).

In the propose model, *U*_*c*_(*x, y*; σ_*u*_), *c* ∈ {*R, G, B, Y*} denotes the response of a subunit centered at (*x, y*) in the RF surround after being inhibited by other subunits. We compute it according to

(8) $$\begin{array}{l} U_{c}(x,y;\sigma_{u})=MAX[0,\;MBP_{c}(x,y)-A_{u}\;\cdot \;MBP_{c}(x,y)\\ \;⊗g(x,y;\sigma_{u})] \end{array}$$

where *A*_*u*_ represents the sensitivity of subunits. *MAX* is a max operator to keep the neuronal response non-negative.

Then, let *S*_*c*_(*x, y*; σ_*s*_), *c* ∈ {*R, G, B, Y*} denote the total responses of all the inhibited subunits in the surround. We compute them as

(9) $$S_{c}(x,y;\sigma_{s})=U_{c}(x,y;\sigma_{u})⊗g(x,y;\sigma_{s}).$$

Then, the final response of a ON ganglion cell, *GC*_*c*_(*x, y*), *c* ∈ {*R, G, B*}, is the response elicited by the excitatory RF center in one channel (e.g., R) subtracted by the total surround inhibition from its opponent channel (e.g., G), which is written as

(10) $$\begin{array}{l} GC_{R}(x,y;\sigma_{cen})=A_{cen}\;\cdot \;MAX[0,MBP_{R}(x,y)⊗g(x,y;\sigma_{cen})\\ \;-A_{s}\;\cdot \;S_{G}(x,y;\sigma_{s})]\\ GC_{G}(x,y;\sigma_{cen})=A_{cen}\;\cdot \;MAX[0,MBP_{G}(x,y)⊗g(x,y;\sigma_{cen})\\ \;-A_{s}\;\cdot \;S_{R}(x,y;\sigma_{s})]\\ GC_{B}(x,y;\sigma_{cen})=A_{cen}\;\cdot \;MAX[0,MBP_{B}(x,y)⊗g(x,y;\sigma_{cen})\\ \;-A_{s}\;\cdot \;S_{Y}(x,y;\sigma_{s})] \end{array}$$

where *A*_*cen*_ represents the sensitivity of the excitatory RF center and *A*_*s*_ is the sensitivity of inhibitory RF surround of ganglion cell.

In Equations (8)–(10), σ_*u*_, σ_*s*_, and σ_*cen*_ are the standard deviations of the 2D Gaussian functions describing the subunit, RF surround and RF center, respectively, and they are set to be one third of the radius of 2D Gaussian shaped regions. In this work, we experimentally set the radius of subunit, RF surround and center as 1, 3, and 1 pixel, respectively. A partial evidence in support of this setting is the neurophysiological finding that the size of RF surround is normally 2–5 times larger (in diameter) than that of RF center (Li and He, 1987; Li et al., 1991, 1992; Li and Li, 1994).

Similarly, with $MBP_{c}^{\prime }\left(x,y\right)$ as the modulated output from a OFF bipolar cell, the response of a OFF ganglion cell, $GC_{c}^{\prime }\left(x,y\right),\;c\in \left\{R,G,B\right\}$, can be easily computed based on Equations (8)–(10).

Biologically, the ON and OFF cells in the retina form almost separated pathways (Schiller, 2010), the signals along which are integrated in the visual cortexes via lateral connections. Modeling such integration is beyond the scope of this work. Here we simply compute the retinal outputs of ON and OFF pathways, respectively, as

(11) $$\begin{array}{l} OutON(x,y)=\frac{1}{3}\sum_{c\in \{R,G,B\}}GC_{c}(x,y)\\ OutOFF(x,y)=\frac{1}{3}\sum_{c\in \{R,G,B\}}GC_{c}^{\prime }(x,y). \end{array}$$

Then the final output of the proposed model is computed by combining the outputs of ON and OFF pathways according to

(12) Out(x,y)=w · OutON(x,y)+(1−w) · (1−OutOFF(x,y))

where *w* is a weight controlling the relative contributions of the ON and OFF pathways. As mentioned above, the ON channel emphasizes the perception of bright regions, and the OFF system emphasizes the perception of dark regions. Such notion will be further validated by our experiment (see the following section). This inspires us to set *w* = *f*_*L*_(*x, y*). Such setting indicates that the signals from the ON and OFF ganglion cells are adaptively combined based on the local brightness at (*x, y*).

## Experiments

In this section, we first show the responsive properties of the model ganglion cells and the model bipolar cells involved in the proposed system to demonstrate how they contribute to the haze removal. We also illustrate the different roles of the ON and OFF pathways in enhancing hazy images. Then we conduct the qualitative comparison on real hazy images as well as quantitative comparison on synthetic images with other representative algorithms.

### Responsive properties of the bipolar cells

**Figure 3:**
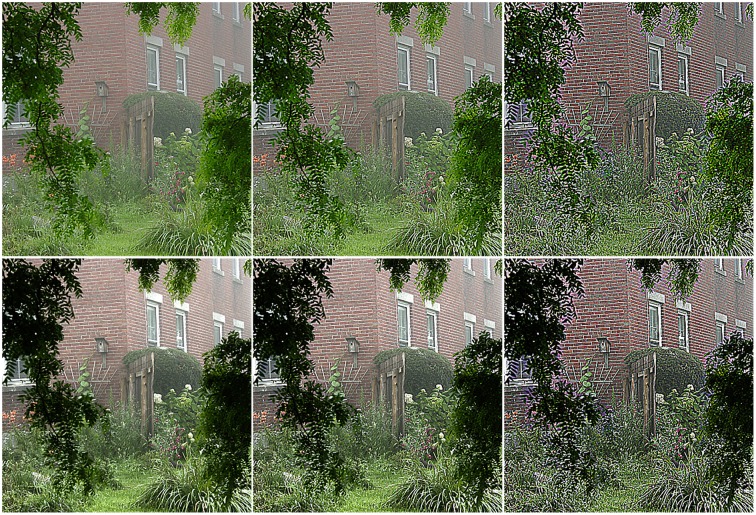
**Response of the bipolar cells**. From left to right, the sensitivity of surround [i.e., the parameter *k* in Equations (2) and (3)] is set to be 0.5, 0.6, and 0.7. The top and bottom rows are the images before and after being modulated by the brightness-driven AII amacrine cells.

The RF of bipolar cell is described by a DOG model [see Equations (3) and (4)]. Due that the DOG-shaped RF is a typical band-pass filter, which may partially attenuate the low frequency components (e.g., the dispersively distributed haze) of the hazy image when the two Gaussian functions are unbalanced. In addition, there is no doubt that the high-frequency details of the image will be also degraded to certain extent. As shown in Figure 3, from left to right, the higher the sensitivity of the inhibitory annular surround [i.e., the parameter *k* in Equations (2) and (3)] is, the greater the bipolar cells remove the haze, but more details loss. The difference between the top and bottom images in Figure 3 shows that the modulation from the rod bipolar cells via AII amacrine cells can compensate the loss of details by increasing the image contrast, but degrade the saturation.

### Responsive properties of the ganglion cells

**Figure 4:**
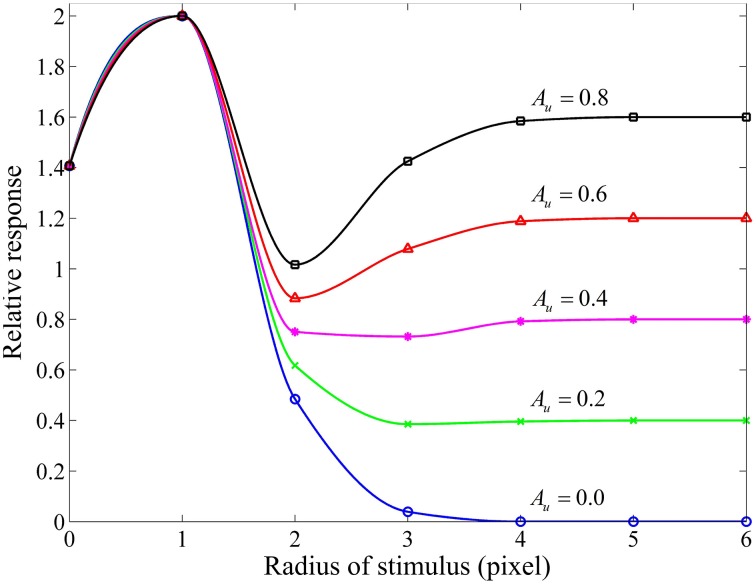
**Responsive curves as a function of stimulus size (in radius) for different subunit sensitivities**. The sizes of RF center, RF surround and subunits are 3, 10, 3 pixels, respectively. The sensitivity of RF surround [i.e., *A*_*s*_ in Equation (9)] is 3.0, and the subunit sensitivity *A*_*u*_ in Equation (8) is set to be 0.8, 0.6, 0.4, 0.2, and 0.0. Relative response represents the final response in channel R (i.e., *GC*_*R*_ (*x, y*)) of a red-ON-green-OFF ganglion cell computed by Equation (10) with *A*_*cen*_ = 1.0 certain *A*_*u*_ when the RF center and its surround are covered by the red and green patches of equi-luminance.

Considering that the RF center and its surround of a red-ON-green-OFF ganglion cell are covered by the red and green patches of equi-luminance, we measured a group of area-response curves (Figure 4) by computing the response of this model ganglion cell with varying subunit sensitivities (*A*_*u*_) while keeping the other parameters fixed. It is clear that for *A*_*u*_ > 0, when the stimulus is extended larger than the RF center, the neuronal response is reduced due to the involved surround inhibition, and then gradually enhanced due to the increasing disinhibition effect deduced by the inhibitory interaction among more surround subunits. This observation is quite consistent with that obtained from electrophysiological studies in the cat retinal ganglion cells (Li and He, 1987; Li et al., 1992; Li and Qiu, 1994). This figure clearly shows that higher *A*_*u*_ values result in stronger disinhibition effects, and hence weaker surround inhibitions and higher neuronal responses.

**Figure 5:**
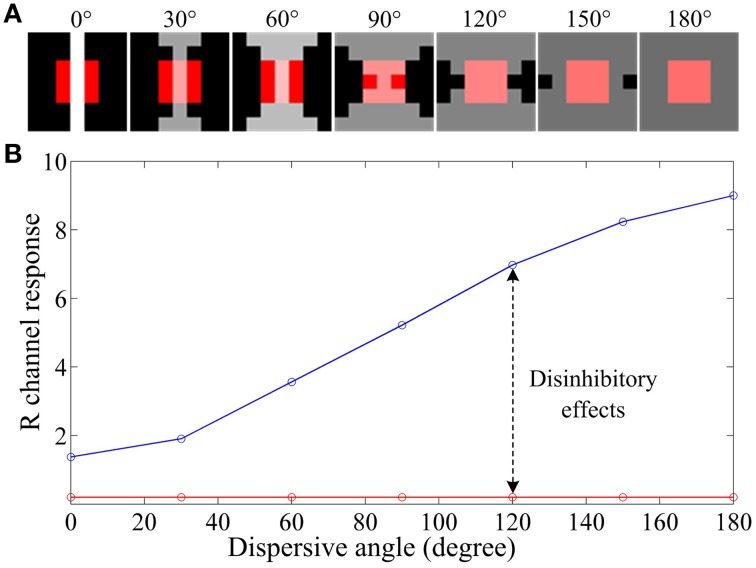
**An example illustrating the effect of various degrees of dispersion of foreground light on the response of a retinal ganglion cell**. **(A)** The stimuli with a size of 5 by 5 pixels, which contains a red block of 3 by 3 pixels covering the full RF center and a patterned bright foreground across both the RF center and its surround. The bright patterns have the same total light flux but different dispersive angles, from 0 to 180°, as indicated on the top of the panels. **(B)** Response vs. dispersion curves of a ganglion cell. The red curve is with surround inhibition but without subunit disinhibition. The blue curve is for the case that both the subunit disinhibition and surround inhibition work. The sizes of RF center, RF surround and subunits are 3^*^3, 7^*^7, 3^*^3 pixels, respectively, with *A*_*cen*_ = 1.0 and *A*_*u*_ = 0.5*A*_*s*_. R channel response denotes *GC*_*R*_(*x, y*) computed by Equation (10) for a red-ON ganglion cell to certain stimulus patch listed in **(A)**.

To illustrate how ganglion cells enhance the hazy images, we designed a series of stimulus patches (Figure 5A), each of which contains a red block (simulating an object surface) covering the full RF center and a patterned white foreground across both the RF center and its black surround. When all parameters are fixed and the white foreground is spatially distributed with systematically increasing dispersion (quantified by a “dispersive angle,” Li and He, 1987; Li et al., 1992; Li and Qiu, 1994) while keeping the total light flux identical, the response of a red-ON ganglion cell to the composited stimuli is consistently increased with the increasing dispersive angle of the white foreground (the blue curve in Figure 5B), because more subunits in the surround are activated and hence stronger disinhibition (and then weaker surround inhibition) is introduced. Note that this increase is not observed when no disinhibition is involved (i.e., *A*_*u*_ = 0), because the surround inhibition is always equal due to the identical total light flux (with any degree of dispersion) in the surround.

Considering that the white foreground also covers the red block in the RF center, more dispersive the white foreground is, more likely it looks that the foreground is the haze (e.g., the last panel of Figure 5A). In this situation, the hazy appearance of the red block (most likely to be a local object surface) is relatively weakened by the increasing of the R channel response, i.e., the red block becomes redder than the original block covered with gray foreground. This example provides a biological explanation about how the disinhibition among subunits in the RF surround contributes to the hazy image enhancement at the retinal ganglion layer.

### Different roles of ON and OFF channels

**Figure 6:**
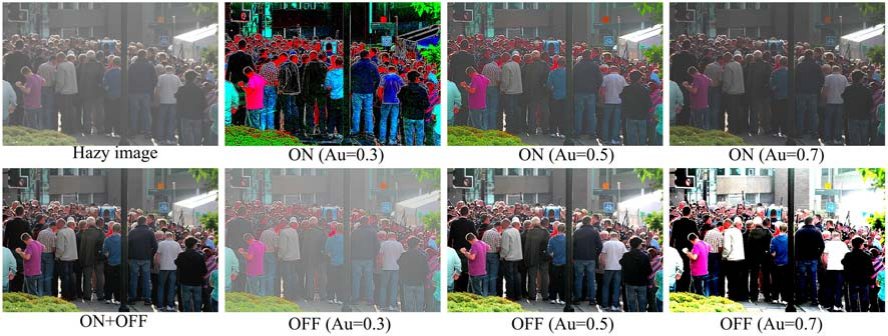
**Illustration of the different roles of ON and OFF pathways in haze removal**. The first image of the top row is the original hazy image. The images from the second to fourth columns of the top and bottom rows are respectively the outputs of ON and OFF ganglion cells with various subunit sensitivity values (*A*_*u*_). The first image of the bottom row is the image after haze removal by the full model proposed in this work, i.e., by linearly combining the output of the ON ganglion cells with *A*_*u*_ = 0.7 and the output of the OFF ganglion cells with *A*_*u*_ = 0.5.

As mentioned above, ON and OFF neurons are excited by the increment and decrement of light intensity in the visual scene, which have been suggested to enable more efficient encoding of sensory stimuli (Joselevitch, 2008). In specific, the functional significance of the separated ON and OFF channels mainly includes the extension of dynamic range, improved SNR, increased spatial resolution, and other information processing benefits (Joselevitch, 2008). For example, ON and OFF bipolar cells would each amplify half of the dynamic range of the photoreceptor signals by responding mainly to the positive and negative contrasts, respectively (Ratliff et al., 2010). Figure 6 shows an example illustrating the different functional roles of the ON and OFF system. For the ON pathway, the quite bright roof of the small tabernacle at the right-top corner of the image can be clearly responded by the ON ganglion cells with appropriate setting (e.g., *A*_*u*_ = 0.5 or 0.7), but the details and colors of the sunless regions (e.g., the dominant region with a crowd of people) can not be properly enhanced along the ON pathway. In contrast, though the OFF system outputs an over-saturated roof of the tabernacle in terms of brightness, this pathway can vividly enhance the details of the dim regions. In short, it is clear that the OFF system carries on more dark elements while the ON system transmits and processes more bright elements. By merging the outputs from the ON and OFF systems, the final result combines the benefits from the both sources, as indicated by the first image of the bottom row in Figure 6.

### Visual comparison on real-world images

For all the input hazy images in the following sections, we experimentally set *k* = 0.3, *A*_*c*_ = *A*_*s*_ = 2, and *A*_*u*_ = 0.7 and 0.5 for the ON and OFF ganglion cells, respectively.

**Figure 7:**
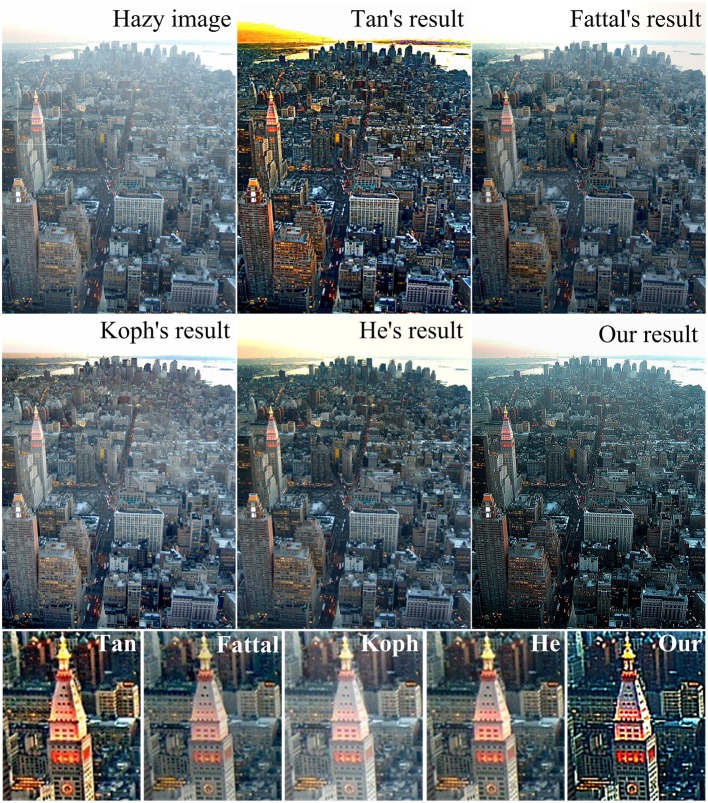
**Qualitative comparison with several representative methods on a real-world image**.

Figure 7 compares the result of our method with that of several state-of-the-art methods (Fattal, 2008; Kopf et al., 2008; Tan, 2008; He et al., 2009, 2011). Note that the results of Tan, Fattal, Koph, and He are quoted from http://research.microsoft.com/en-us/um/people/kahe/. In specific, Tan's method (Tan, 2008) removes haze by maximizing the local contrast of the input hazy image, which can well-enhance the local contrast, but normally results in oversaturated colors and halo artifacts. Fattal's method (Fattal, 2008) eliminates the scattered light by estimating the optical transmission in hazy scenes and requires sufficient color information and variance. Considering that the color of distant parts of the hazy image in Figure 7 is faint, which does not satisfy the requirement of Fattal's method, and hence in his result, this part is too bright to see the details like the towers. The method of Kopf et al. (2008) removes the haze by 3D models and texture maps of the scene, which needs additional geography information. With visible residual hazes, the result image of this method is not very clear. The method of He et al. (2009, 2011) is based on the dark channel prior. By removing haze and enhancing details, our result is comparable in saturation with He's, but has higher visibility of structures, except a little haze left over the distant region.

**Figure 8:**
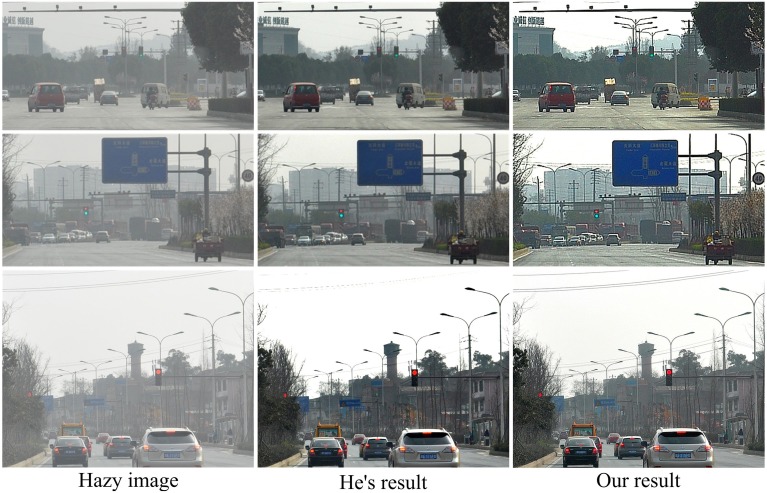
**Comparisons with He's method on real-world traffic images**.

In Figure 8, we also compare our method with He's method on several traffic scenes captured by our group. Note that He's results on these images were calculated by us with the common parameter setting suggested in their paper: ω = 0.9, *t*_0_ = 0.1 and a patch size of 15^*^15 pixels (see the detailed meaning of these parameters in He et al., 2009, 2011). Since He's dark channel prior based method estimates the atmospheric light (the parameter A in his model) as constant, its result is sometimes a little dark and may lose some details (e.g., the electrical wires occurring in the sky region of the bottom image of Figure 8) when the atmospheric light seems to vary spatially. In contrast, in the results recovered by our model, cars are easier to see, and the road markings and traffic signs are more visible and can be easier to read out.

### Quantitative comparison on synthetic images

With the haze-free images and the known disparity maps *d*(*x*) (Scharstein and Szeliski, 2003; Hirschmuller and Scharstein, 2007; Scharstein and Pal, 2007), we set the transmission map *t*(*x*) = 0.8 × *d*(*x*) in the Koschmieder model (Koschmieder, 1925), and then synthesize the hazy images according to

(13) I(x)=J(x) · t(x)+A · (1−t(x))

where *J*(*x*) is the input haze-free image, *I*(*x*) is the hazy image, and *A* is the global atmospheric airlight, assumed as pure white for simplicity.

**Figure 9:**
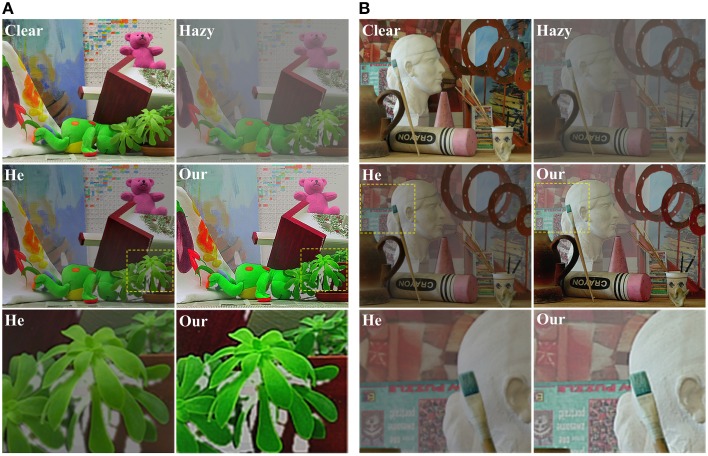
**Comparison with He's method on two synthetic hazy images**. **(A)** *Teddy* image, **(B)** *Art* image. For each of **(A,B)**, the top row shows the clear image and the image added with haze, the middle row lists the results by He's and our methods, and the bottom row lists the enlarged local patches. The *Teddy* and *Art* images are downloaded from http://vision.middlebury.edu/stereo/data/.

Quantifying by the commonly used mean squared error (MSE) between the original haze-free image and the recovered image, we compared the performance of our method with that of He (He et al., 2009, 2011) in Table 1 on a group of synthetic images. In general, a lower MSE means a better performance in removing haze. This table clearly shows that our results are much more close to the original haze-free images in all eight scenes. Two examples are shown in Figure 9. He's results are a little dark and our results have more vivid colors. The enlarged local patches show that He's results are a little hazier than that by our model.

**Table 1:** **Quantitative comparison of MSE with He's model on synthetic images**.

**Scenes**	**He's result**	**Our result**
Cone	0.0083	0.0038
Teddy	0.0102	0.0040
Art	0.0087	0.0059
Books	0.0099	0.0037
Dolls	0.0062	0.0042
Laundry	0.0077	0.0038
Moebius	0.0048	0.0032
Reindeer	0.0116	0.0092

## Discussion

In this paper, without explicit assumption, we proposed a hazy image enhancement method inspired by the information processing mechanisms of the retinal network in the biological visual system, from the photoreceptors absorption via the bipolar cells to the opponent ganglion cells equipped with subunit-structured RF surround. The proposed model was compared with the state-of-the-art methods qualitatively and quantitatively, and shows competitive results on both real-world and synthetic images. In particular, our model can remove the haze as well as clearly enhance the details, without sacrificing the color fidelity.

This simplified retinal model proposed here also provides valuable suggestions about the role of the RGC surround disinhibition as well as the ON and OFF pathways in image enhancement. As shown in Equation (13), an image with haze loses its contrast and saturation. In our model, the DOG shaped RF of bipolar cells, which partially filters out the low spatial frequency elements, serves to obtain an incomplete but fast reduction in the obvious influence of the airlight components. The modulation from the AII amacrine cells driven by rod bipolars is capable of increasing the contrast of the image, but decreasing the saturation. This undesired decrease of saturation is then compensated by the chromatically single-opponent RGCs. Subunit-deduced surround disinhibition in RGCs contributes a fine and adaptive correction based on the spatial resolution of the disinhibition. Roughly speaking, the proposed model realizes the haze removal by enhancing the contrast of details and recovering the object colors while reducing the low spatial frequency components of scenes.

**Figure 10:**
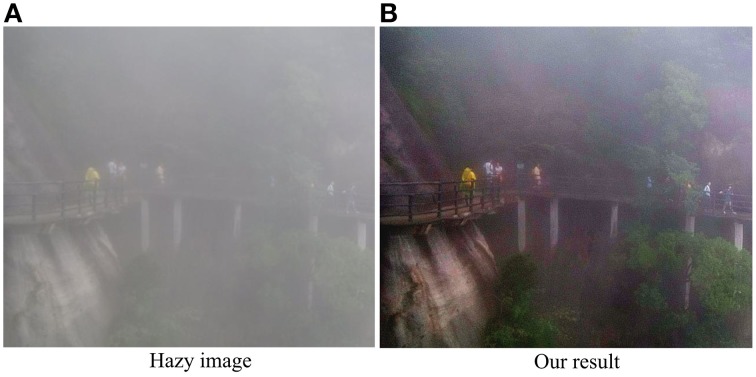
**Our result on an image with dense haze**. **(A)** The hazy image. **(B)** The image after haze removal by the proposed method.

Though performing well in dehazing and enhancing single haze images, our model also has some limitations. In particular, our method does not perform quite well for the images with quite dense haze. As shown in Figure 10, due to the heterogeneously distrusted thick haze, the travelers, trestle and plants are almost visually undetectable. Though the visibility is improved significantly after haze removal by our model, there is still clear haze left in the top part of the scene. However, it is interesting to point out that the dehazing performance for the scenes with heterogeneously distributed heavy haze as shown in Figure 10 may be improved by introducing the dynamic spatiotemporal structure of receptive field (RF). In the space domain, profound contrast-dependent change in RF size, normally an inverse relationship between stimulus contrast and the RF size, has been well-observed for the retinal ganglion cells, LGN and V1 cells (Sceniak et al., 1999; Nolt et al., 2004; Chen et al., 2013). For example, at the level of retina, Nolt et al. (2004) reported that the size of the receptive field center decreases with an increase in contrast for both LGN cells and RGCs. In particular, the center size was, on average, 1.99 times greater at low contrast than at high contrast for the RGCs. Such adaptive changes in the spatial summation as a function of local contrast may allow the visual system to optimize performance under changing stimulus conditions (Sceniak et al., 1999). For example, for the heavy hazy local regions of low contrast, expansion of spatial summation may produce increased sensitivity and a better detection capability for weak signals (with the sacrificing of spatial resolution) of an image. Inspired by such property of contrast-dependent change in RF size and the results shown in Figure 6 for the analysis of the roles of ON and OFF systems, we expect to improve the performance of our model in the future by automatically selecting the model parameters that are spatially adaptive to the local haze related visual features.

Undoubtedly, another future direction is to introduce the information processing mechanisms of the higher visual cortexes, considering that the scene depth is a quite important cue that is closely related to the thickness of haze. Based on the binocular disparity, the human visual system can easily extract the scene depth (Haefner and Cumming, 2007), which is expected to be especially helpful for the observers to estimate and remove the haze before the objects, though it is very difficult for the visual system to exactly extract the binocular disparity from the scenes with heterogeneously distributed thick haze.

## Author contributions

XZ conducted the experiments and wrote the paper. SG conducted the experiments. CL reviewed and edited the manuscript. YL designed and supervised the study and reviewed and edited the manuscript.

### Conflict of interest statement

The authors declare that the research was conducted in the absence of any commercial or financial relationships that could be construed as a potential conflict of interest.

## References

[B1] Alahi A. Ortiz R. Vandergheynst P. (2012). Freak: fast retina keypoint, in IEEE Conference on Computer Vision and Pattern Recognition (CVPR) (Providence, RI), 510–517.

[B2] Ancuti C. O. Ancuti C. Hermans C. Bekaert P. (2011). A fast semi-inverse approach to detect and remove the haze from a single image, in Computer Vision–ACCV (Queenstown), 501–514.

[B3] Buzás P. Blessing E. M. Szmajda B. A. Martin P. R. (2006). Specificity of M and L cone inputs to receptive fields in the parvocellular pathway: random wiring with functional bias. J. Neurosci. 26, 11148–11161. 10.1523/JNEUROSCI.3237-06.2006 17065455 PMC6674646

[B4] Caraffa L. Tarel J.-P. (2013). Markov random field model for single image defogging, in IEEE Intelligent Vehicles Symposium (IV) (Gold Coast, QLD), 994–999.

[B5] Chen K. Song X. M. Li C. Y. (2013). Contrast-dependent variations in the excitatory classical receptive field and suppressive nonclassical receptive field of cat primary visual cortex. Cereb. Cortex 23, 283–292. 10.1093/cercor/bhs012 22302117

[B6] Chen S. Li W. (2012). A color-coding amacrine cell may provide a blue-off signal in a mammalian retina. Nat. Neurosci. 15, 954–956. 10.1038/nn.3128 22634731 PMC3386466

[B7] Conway B. R. Chatterjee S. Field G. D. Horwitz G. D. Johnson E. N. Koida K.. (2010). Advances in color science: from retina to behavior. J. Neurosci. 30, 14955–14963. 10.1523/JNEUROSCI.4348-10.2010 21068298 PMC3073527

[B8] Crook J. D. Manookin M. B. Packer O. S. Dacey D. M. (2011). Horizontal cell feedback without cone type-selective inhibition mediates “red–green” color opponency in midget ganglion cells of the primate retina. J. Neurosci. 31, 1762–1772. 10.1523/JNEUROSCI.4385-10.2011 21289186 PMC3074339

[B9] Enroth-Cugell C. Robson J. G. (1966). The contrast sensitivity of retinal ganglion cells of the cat. J. Physiol. 187, 517–552. 10.1113/jphysiol.1966.sp008107 16783910 PMC1395960

[B10] Euler T. Haverkamp S. Schubert T. Baden T. (2014). Retinal bipolar cells: elementary building blocks of vision. Nat. Rev. Neurosci. 15, 507–519. 10.1038/nrn3783 25158357

[B11] Fattal R. (2008). Single image dehazing, in ACM Transactions on Graphics (TOG): ACM (Los Angeles, CA), 72 10.1145/1399504.1360671

[B12] Field G. D. Gauthier J. L. Sher A. Greschner M. Machado T. A. Jepson L. H.. (2010). Functional connectivity in the retina at the resolution of photoreceptors. Nature 467, 673–677. 10.1038/nature09424 20930838 PMC2953734

[B13] Foster D. H. (2011). Color constancy. Vision Res. 51, 674–700. 10.1016/j.visres.2010.09.006 20849875

[B14] Gao S. B. Yang K. F. Li C. Y. Li Y. J. (2013). A color constancy model with double-opponency mechanisms, in IEEE International Conference on Computer Vision (ICCV) (Sydney), 929–936.

[B15] Gao S. B. Yang K. F. Li C. Y. Li Y. J. (2015). Color constancy using double-opponency. IEEE Trans. Pattern Anal. Mach. Intell. 37, 1973–1985. 10.1109/TPAMI.2015.2396053 26353182

[B16] Ghassemian H. (2001). A retina based multi-resolution image-fusion, in Geoscience and Remote Sensing Symposium, 2001. IGARSS'01. IEEE 2001 International (Sydney), 709–711.

[B17] Gibson K. B. Võ D. T. Nguyen T. Q. (2012). An investigation of dehazing effects on image and video coding. IEEE Trans. Image Process. 21, 662–673. 10.1109/TIP.2011.2166968 21896391

[B18] Gollisch T. Meister M. (2010). Eye smarter than scientists believed: neural computations in circuits of the retina. Neuron 65, 150–164. 10.1016/j.neuron.2009.12.009 20152123 PMC3717333

[B19] Haefner R. M. Cumming B. G. (2007). Adaptation to natural binocular disparities in primate V1 explained by a generalized energy model. Neuron 57, 147–158. 10.1016/j.neuron.2007.10.042 18184571 PMC2344156

[B20] Hautière N. Tarel J.-P. Aubert D. (2007). Towards fog-free in-vehicle vision systems through contrast restoration, in IEEE Conference on Computer Vision and Pattern Recognition, CVPR (Minneapolis, MN), 1–8.

[B21] He K. Sun J. Tang X. (2009). Single image haze removal using dark channel prior, in IEEE Conference on Computer Vision and Pattern Recognition, CVPR (Miami, FL), 1956–1963. 10.1109/TPAMI.2010.168 20820075

[B22] He K. Sun J. Tang X. (2011). Single image haze removal using dark channel prior. IEEE Trans. Pattern Anal. Mach. Intell. 33, 2341–2353. 10.1109/TPAMI.2010.168 20820075

[B23] Hirschmuller H. Scharstein D. (2007). Evaluation of cost functions for stereo matching, in IEEE Conference on Computer Vision and Pattern Recognition, CVPR'07 (Minneapolis, MN), 1–8.

[B24] Jobson D. J. Rahman Z.-U. Woodell G. A. (1997). A multiscale retinex for bridging the gap between color images and the human observation of scenes. IEEE Trans. Image Process. 6, 965–976. 10.1109/83.597272 18282987

[B25] Joselevitch C. (2008). Human retinal circuitry and physiology. Psychol. Neurosci. 1, 141–165. 10.3922/j.psns.2008.2.008

[B26] Kaneko A. Tachibana M. (1983). Double color-opponent receptive fields of carp bipolar cells. Vision Res. 23, 381–388. 10.1016/0042-6989(83)90085-8 6880036

[B27] Kopf J. Neubert B. Chen B. Cohen M. Cohen-Or D. Deussen O. (2008). Deep photo: Model-based photograph enhancement and viewing, in ACM Transactions on Graphics (TOG) (Providence, RI).

[B28] Koschmieder H. (1925). Theorie der Horizontalen Sichtweite: Kontrast und Sichtweite. Munich: Keim & Nemnich.

[B29] Kratz L. Nishino K. (2009). Factorizing scene albedo and depth from a single foggy image, in IEEE 12th International Conference on Computer Vision (Kyoto), 1701–1708. 10.1109/iccv.2009.5459382

[B30] Land E. H. (1986). Recent advances in retinex theory. Vision Res. 26, 7–21. 10.1016/0042-6989(86)90067-2 3716215

[B31] Land E. H. McCann J. (1971). Lightness and retinex theory. J. Opt. Soc. Am. 61, 1–11. 10.1364/JOSA.61.000001 5541571

[B32] Lee B. B. Martin P. R. Grünert U. (2010). Retinal connectivity and primate vision. Prog. Retin. Eye Res. 29, 622–639. 10.1016/j.preteyeres.2010.08.004 20826226 PMC3282052

[B33] Lee B. B. Shapley R. M. Hawken M. J. Sun H. (2012). Spatial distributions of cone inputs to cells of the parvocellular pathway investigated with cone-isolating gratings. J. Opt. Soc. Am. A Opt.Image Sci. Vis. 29, A223–A232. 10.1364/JOSAA.29.00A223 22330383 PMC4237200

[B34] Li C. Y. (1996). Integration fields beyond the classical receptive field: organization and functional properties. News Physiol. Sci. 11, 181–186.

[B35] Li C. Y. He Z. J. (1987). Effects of patterned backgrounds on responses of lateral geniculate neurons in cat. Exp. Brain Res. 67, 16–26. 10.1007/BF00269448 3622676

[B36] Li C. Y. Li W. (1994). Extensive integration field beyond the classical receptive field of cat's striate cortical neurons—classification and tuning properties. Vision Res. 34, 2337–2355. 10.1016/0042-6989(94)90280-1 7975275

[B37] Li C. Y. Pei X. Zhow Y. X. von Mitzlaff H. C. (1991). Role of the extensive area outside the X-cell receptive field in brightness information transmission. Vision Res. 31, 1529–1540. 10.1016/0042-6989(91)90130-W 1949622

[B38] Li C. Y. Qiu F. T. (1994). Simulation of spatial transfer properties of cat retinal ganglion cell. Acta Biophys. Sin. 11, 395–400.

[B39] Li C. Y. Zhou Y. X. Pei X. Qiu F. T. Tang C. Q. Xu X. Z. (1992). Extensive disinhibitory region beyond the classical receptive field of cat retinal ganglion cells. Vision Res. 32, 219–228. 10.1016/0042-6989(92)90131-2 1574837

[B40] Martin P. R. Lee B. B. White A. J. Solomon S. G. Rüttiger L. (2001). Chromatic sensitivity of ganglion cells in the peripheral primate retina. Nature 410, 933–936. 10.1038/35073587 11309618

[B41] Masland R. H. (2012). The neuronal organization of the retina. Neuron 76, 266–280. 10.1016/j.neuron.2012.10.002 23083731 PMC3714606

[B42] Meng G. Wang Y. Duan J. Xiang S. Pan C. (2013). Efficient image dehazing with boundary constraint and contextual regularization, in IEEE International Conference on Computer Vision (ICCV) (Sydney), 617–624.

[B43] Mills S. L. Tian L.-M. Hoshi H. Whitaker C. M. Massey S. C. (2014). Three distinct blue-green color pathways in a mammalian retina. J. Neurosci. 34, 1760–1768. 10.1523/JNEUROSCI.3901-13.2014 24478358 PMC3905144

[B44] Nair D. Kumar P. A. Sankaran P. (2014). An effective surround filter for image dehazing, in Proceedings of the 2014 International Conference on Interdisciplinary Advances in Applied Computing (New York, NY).

[B45] Namer E. Shwartz S. Schechner Y. Y. (2009). Skyless polarimetric calibration and visibility enhancement. Opt. Express 17, 472–493. 10.1364/OE.17.000472 19158860

[B46] Narasimhan S. G. Nayar S. K. (2000). Chromatic framework for vision in bad weather, in Proceedings. IEEE Conference on Computer Vision and Pattern Recognition, CVPR (Hilton Head Island, SC), 598–605.

[B47] Narasimhan S. G. Nayar S. K. (2003a). Contrast restoration of weather degraded images. IEEE Trans. Patt. Anal. Mach. Intell. 25, 713–724. 10.1109/TPAMI.2003.1201821

[B48] Narasimhan S. G. Nayar S. K. (2003b). Interactive (de) weathering of an image using physical models, in IEEE Workshop on Color and Photometric Methods in Computer Vision (Nice).

[B49] Nayar S. K. Narasimhan S. G. (1999). Vision in bad weather, in The Proceedings of the Seventh IEEE International Conference on Computer Vision (Kerkyra), 820–827.

[B50] Nolt M. J. Kumbhani R. D. Palmer L. A. (2004). Contrast-dependent spatial summation in the lateral geniculate nucleus and retina of the cat. J. Neurophysiol. 92, 1708–1717. 10.1152/jn.00176.2004 15128751

[B51] Qiu F. T. Li C. Y. (1995). Mathematical simulation of disinhibitory properties of concentric receptive field. Acta Biophys. Sin. 11, 214–220.

[B52] Rahman Z.-U. Jobson D. J. Woodell G. A. (2004). Retinex processing for automatic image enhancement. J. Electron. Imaging 13, 100–110. 10.1117/1.1636183

[B53] Rajput G. S. Rahman Z. U. (2008). Hazard detection on runways using image processing techniques, in SPIE Defense and Security Symposium: International Society for Optics and Photonics), 69570D–69512.

[B54] Ratliff C. P. Borghuis B. G. Kao Y.-H. Sterling P. Balasubramanian V. (2010). Retina is structured to process an excess of darkness in natural scenes. Proc. Natl. Acad. Sci. U.S.A. 107, 17368–17373. 10.1073/pnas.1005846107 20855627 PMC2951394

[B55] Reid R. C. Shapley R. M. (2002). Space and time maps of cone photoreceptor signals in macaque lateral geniculate nucleus. J. Neurosci. 22, 6158–6175. 12122075 10.1523/JNEUROSCI.22-14-06158.2002 PMC6757940

[B56] Rodieck R. W. Stone J. (1965). Response of cat retinal ganglion cells to moving visual patterns. J. Neurophysiol. 28, 819–832. 5867881 10.1152/jn.1965.28.5.819

[B57] Sceniak M. P. Ringach D. L. Hawken M. J. Shapley R. (1999). Contrast's effect on spatial summation by macaque V1 neurons. Nat. Neurosci. 2, 733–739. 10.1038/11197 10412063

[B58] Scharstein D. Pal C. (2007). Learning conditional random fields for stereo, in IEEE Conference on Computer Vision and Pattern Recognition, CVPR'07 (Minneapolis, MN), 1–8.

[B59] Scharstein D. Szeliski R. (2003). High-accuracy stereo depth maps using structured light, in Proceedings. 2003. IEEE Computer Society Conference on Computer Vision and Pattern Recognition (Madison, WI), I-195–I-202.

[B60] Schaul L. Fredembach C. Süsstrunk S. (2009). Color image dehazing using the near-infrared, in IEEE International Conference on Image ProcessingCiteseer (Cairo), 1629–1632.

[B61] Schechner Y. Y. Narasimhan S. G. Nayar S. K. (2001). Instant dehazing of images using polarization, in Proceedings of the 2001 IEEE Computer Society Conference on Computer Vision and Pattern Recognition, 2001. CVPR 2001 (Kauai), I-325–I-332.

[B62] Schiller P. H. (2010). Parallel information processing channels created in the retina. Proc. Natl. Acad. Sci. U.S.A. 107, 17087–17094. 10.1073/pnas.1011782107 20876118 PMC2951406

[B63] Shwartz S. Namer E. Schechner Y. Y. (2006). Blind haze separation, in Computer Vision and Pattern Recognition, 2006. CVPR 2006. IEEE Computer Society Conference on, 1984–1991. 10.1109/cvpr.2006.71

[B64] Spitzer H. Barkan Y. (2005). Computational adaptation model and its predictions for color induction of first and second orders. Vision Res. 45, 3323–3342. 10.1016/j.visres.2005.08.002 16169037

[B65] Spitzer H. Semo S. (2002). Color constancy: a biological model and its application for still and video images. Patt. Recogn. 35, 1645–1659. 10.1016/S0031-3203(01)00160-1

[B66] Sun H. Smithson H. E. Zaidi Q. Lee B. B. (2006). Specificity of cone inputs to macaque retinal ganglion cells. J. Neurophysiol. 95, 837–849. 10.1152/jn.00714.2005 16424455 PMC2843159

[B67] Tan K. Oakley J. P. (2000). Enhancement of color images in poor visibility conditions, in Image Processing, 2000. Proceedings. 2000 International Conference on (Vancouver, BC), 788–791.

[B68] Tan R. T. (2008). Visibility in bad weather from a single image, in Computer Vision and Pattern Recognition, 2008. CVPR 2008. IEEE Conference on (Anchorage, AK), 1–8. 10.1109/cvpr.2008.4587643

[B69] Tang K. Yang J. Wang J. (2014). Investigating haze-relevant features in a learning framework for image dehazing, in Computer Vision and Pattern Recognition, 2014. CVPR 2014. IEEE Conference on (Columbus, OH), 2995–3002. 10.1109/cvpr.2014.383

[B70] Tarel J.-P. Hautiere N. (2009). Fast visibility restoration from a single color or gray level image, in Computer Vision, 2009 IEEE 12th International Conference on (Kyoto), 2201–2208. 10.1109/iccv.2009.5459251

[B71] Tarel J.-P. Hautière N. Caraffa L. Cord A. Halmaoui H. Gruyer D. (2012). Vision enhancement in homogeneous and heterogeneous fog. IEEE Intell. Transp. Syst. Mag. 4, 6–20. 10.1109/MITS.2012.2189969

[B72] Thoreson W. B. Mangel S. C. (2012). Lateral interactions in the outer retina. Prog. Retin. Eye Res. 31, 407–441. 10.1016/j.preteyeres.2012.04.003 22580106 PMC3401171

[B73] Treibitz T. Schechner Y. Y. (2009). Polarization: beneficial for visibility enhancement?, in Computer Vision and Pattern Recognition, 2009. CVPR 2009. IEEE Conference on (Miami, FL), 525–532. 10.1109/cvpr.2009.5206551

[B74] Vu N.-S. Caplier A. (2009). Illumination-robust face recognition using retina modeling, in Image Processing (ICIP), 2009 16th IEEE International Conference on (Cairo), 3289–3292.

[B75] Werblin F. S. Dowling J. E. (1969a). Organization of the retina of the mudpuppy, necturus macubsus. II. Intracellular recording. J. Neurophysiol. 32, 339. 4306897 10.1152/jn.1969.32.3.339

[B76] Werblin F. S. Dowling J. E. (1969b). Organization of the retina of the mudpuppy, Necturus maculosus. II. Intracellular recording. J. Neurophysiol. 32, 339–355. 4306897 10.1152/jn.1969.32.3.339

[B77] Woodell G. Jobson D. J. Rahman Z.-u. Hines G. (2005). Enhancement of imagery in poor visibility conditions, in Defense and Security: International Society for Optics and Photonics (Orlando, FL), 673–683.

[B78] Xie B. Guo F. Cai Z. (2010). Improved single image dehazing using dark channel prior and multi-scale Retinex, in Intelligent System Design and Engineering Application (ISDEA), 2010 International Conference on (Hong Kong), 848–851. 10.1109/isdea.2010.141

[B79] Yang K. F. Gao S. B. Guo C. F. Li C. Y. Li Y. J. (2015). Boundary detection using double-opponency and spatial sparseness constraint. IEEE Trans. Image Process. 24, 2565–2578. 10.1109/TIP.2015.2425538 25910090

[B80] Yang K. F. Gao S. B. Li C. Y. Li Y. J. (2013). Efficient color boundary detection with color-opponent mechanisms, in Computer Vision and Pattern Recognition (CVPR), 2013 IEEE Conference on (Portland, OR), 2810–2817. 10.1109/cvpr.2013.362

[B81] Yu J. Xiao C. Li D. (2010). Physics-based fast single image fog removal, in Signal Processing (ICSP), 2010 IEEE 10th International Conference on (Beijing), 1048–1052. 10.1109/icosp.2010.5655901

[B82] Zhou J. Zhou F. (2013). Single image dehazing motivated by Retinex theory, in Instrumentation and Measurement, Sensor Network and Automation (IMSNA), 2013 2nd International Symposium on (Toronto, ON), 243–247. 10.1109/imsna.2013.6743260

